# Vomissements associés à une stagnation pondérale et convulsions: penser à une anomalie du cycle d’urée

**DOI:** 10.11604/pamj.2018.31.103.11403

**Published:** 2018-10-10

**Authors:** Brahim El Hasbaoui, Saloua Boujrad, Rachid Abilkacem, Aomar Agadr

**Affiliations:** 1 Service de Pédiatrie, Hôpital Militaire d’Instruction Mohammed V, Faculté de Médecine et de Pharmacie, Université Mohammed V, Rabat, Maroc

**Keywords:** Anomalie du cycle de l´urée, vomissement, stagnation pondérale, hyperammoniémie, Urea cycle disorder, vomiting, weight stagnation, hyperammonemia

## Abstract

Dans certaines maladies métaboliques héréditaires, les vomissements peuvent apparaître comme un symptôme étant au premier plan, en particulier les anomalies du cycle de l'urée, qui sont habituellement diagnostiqués en période néonatale ou dans l'enfance. Nous en rapportons un cas de révélation tardive par un état de mal convulsif. Nous rapportons le cas d'une patiente âgée de 13 ans, qui a été hospitalisé pour prise en charge d'un état de mal convulsif et un retard staturo-pondéral. L'interrogatoire a révélé la notion de vomissements chroniques avec des troubles du comportement, ralentissement idéomoteur et céphalées. L'examen a trouvé une ataxie. La ponction lombaire et le scanner cérébral sont normaux. Une ammoniémie nettement augmentée est mise en évidence 75 micromoles/l (11-50). La chromatographie des acides aminés dans le sang a montré une augmentation de la glutamine et de l'alanine, La chromatographie des acides aminés dans les urines a montré une augmentation des acides aminés basiques évoquant un déficit du cycle de l'urée par déficit de l'enzyme Argininosuccinate lyase. La patiente a été traité en urgence par une alimentation exclusivement glucidolipidique, et par benzoate de sodium permettant une amélioration de l'état clinique, et une reprise de poids. Les crises convulsives ont été maîtrisées par le phénobarbital. L'enquête familiale a trouvé une sœur âgée de 20 ans suivie depuis l'âge de 3 ans pour crises convulsives traité par le phénobarbital dont le bilan métabolique réalisé dans notre service a objectivé la même anomalie du cycle de l'urée que sa sœur. A tout âge, devant une encéphalopathie avec épilepsie, vomissement, stagnation pondérale et hyperammoniémie, il faut penser à un déficit du cycle de l'urée. Le diagnostic est très souvent posé lors d'un accès neuro-digestif aigue associant vomissements, troubles de conscience et/ou crises convulsives.

## Abstract (en)

In some inherited metabolic diseases, in particular in urea cycle disorders, which are usually diagnosed in neonatal period or in childhood, vomiting is often the first symptom. We report a case of late revelation of urea cycle disorder in a 13 years old female patient hospitalized for convulsions and failure to thrive. The patient underwent an interview revealing chronic vomiting associated with behavioral disorders, ideomotor slowdown and headaches. Clinical examination showed ataxia. Lumbar puncture and head CT scan were normal. The patient had substantially elevated blood ammonia level at 75 micromoles/L (11-50). Chromatography of amino acids in the blood showed increased glutamine and alanine. Chromatography of amino acids in the urine showed increased basic amino acids evoking a deficit of the urea cycle due to deficit of the enzyme argininosuccinate lyase. The patient was treated as an emergency, exclusively with glycolipid-diet and sodium benzoate, allowing improvement of patient’s clinical condition and weight resumption. The seizures were controlled by phenobarbital. Family interview revealed that patient’s sister, aged 20 years, had a 3-year history of seizures treated with phenobarbital. She underwent metabolic assessment in our department, which showed the same urea cycle abnormality as hes sister. Urea cycle deficiency should be suspected in patients of any age with encephalopathy associated with epilepsy, vomiting, weight stagnation and hyperammonemia. The diagnosis is very often made during severe neurodigestive attack involving vomiting, attack and/or seizures.

## Introduction

Les déficits du cycle de l'urée (urea cycle disorders, UCD) sont des maladies héréditaires du métabolisme dont les signes cliniques sont en partie dus à une intoxication liée à une hyperammoniémie et à une élévation des acides aminés transporteurs d'ammoniac. Les UCDs ne sont pas exclusivement des maladies de la période néonatale ou de la petite enfance car le diagnostic peut être établi à tout âge [1]. Le déficit en ornithine transcarbamylase (OTC) est l'UCD le plus fréquent [1, 2], et celui dont la sémiologie est la mieux décrite, y compris chez l'adulte [3]. Les décompensations métaboliques aiguës sont potentiellement mortelles.

## Patient et observation

Une patiente de 13 ans, sans antécédents pathologiques notables, elle n'a pas de consanguinité parentale, un poids de 30 kg (-2DS) pour une taille de 1,47 m (-2DS), elle a été hospitalisé dans notre formation pour des vomissements chroniques réfractaire au traitement symptomatique et qui se sont associés par la suite a de troubles de comportement, un ralentissement idéomoteur et des céphalées. L'examen clinique n'a pas trouvé de déficit neurologique, mais a montré une ataxie. Au cours de son hospitalisation l'enfant a présenté un état de mal épileptique ayant nécessité un séjour en réanimation. Sur le plan paraclinique : la ponction lombaire et le scanner cérébral étaient normaux. L'ammoniémie est augmentée : 75 micromoles/l (11-50). La chromatographie des acides aminés dans le sang a montré une augmentation de la Glutamine et de l'alanine, La chromatographie des acides aminés dans les urines a montré une augmentation des acides aminés basiques (acide argininisuccinique) évoquant un déficit du cycle de l'urée par déficit de l'enzyme Argininosuccinate lyase. La patiente a été traité en urgence par une alimentation exclusivement glucidolipidique, et par benzoate de sodium permettant une amélioration de l'état clinique, et une reprise de poids. Les crises convulsives ont été maitrisées par le phénobarbital. L'enquête familiale a trouvé une sœur âgée de 20 ans suivie depuis l'âge de 3 ans pour crises convulsives gérer par le phénobarbital et dont le bilan métabolique réaliser dans notre service à objectivé la même anomalie du cycle de l'urée que sa sœur.

## Discussion

**Figure 1:**
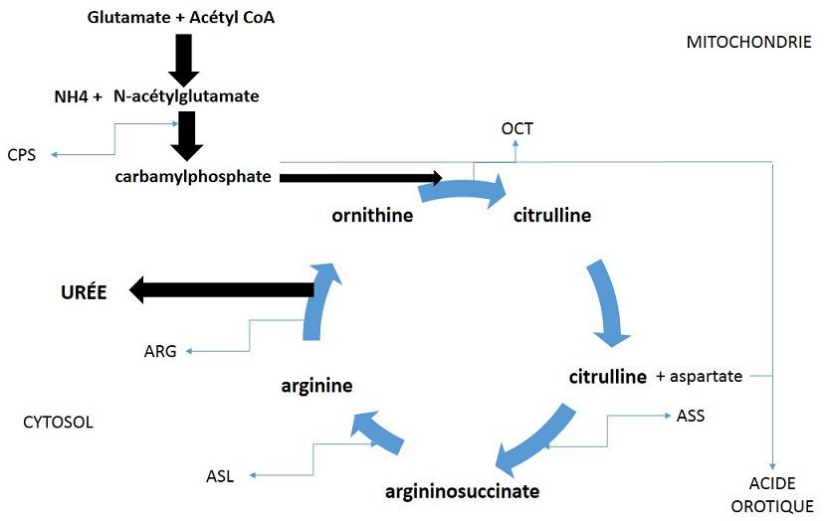
Cycle de l’urée: NAGS: N-acétyl glutamate synthétase; CPS: carbamylphopshate synthétase; OCT: ornithine car- bamyl transferase; ASS: argininosuccinate synthétase; ASL: argininosuccinate lyase; ARG: arginase

L'ammoniac est issu du métabolisme de la glutamine dans les cellules intestinales et rénales, et de l'alanine qui est l'un des substrats du métabolisme musculaire. L'ammoniac provient également de la désamination intra-hépatique de certains acides aminés comme la glutamine, l'asparagine, la sérine, la thréonine, la glycine. Il existe 2 systèmes de détoxification de l'ammoniac au niveau hépatique [4]. Le premier prédomine au niveau des hépatocytes péri-veineux par la synthèse de glutamine sous l'action de la glutamine synthétase. Le second est le cycle de l'urée qui permet d'éliminer l'excès d'azote sous forme d'urée hydrosoluble éliminée par voie urinaire. Le cycle de l'urée a lieu dans les hépatocytes péri-portaux et comprend 6 réactions enzymatiques successives. La N-acétylglutamate synthétase (NAGS), la carbamylphosphate synthétase (CPS) et l'ornithine transcarbamylase (OTC) sont les 3 enzymes mitochondriales du cycle de l'urée, alors que l'argininosuccinate synthétase (ASS), l'argininosuccinatelyase (ASL) et l'arginase sont cytoplasmiques (Figure 1). Un déficit de chacune de ces enzymes peut conduire à une accumulation de précurseurs en amont de l'enzyme déficiente et une carence des métabolites en aval avec pour résultat un œdème cérébral d'origine osmotique et une encéphalopathie à l'origine de troubles neuropsychiatriques [5]. La recherche d'une sélection dans l'alimentation orientée vers une alimentation végétarienne est un critère important. En effet, ces patients rapportent fréquemment une intolérance ou un dégoût pour une alimentation riche en protéines car responsable d'accès d'hyperammoniémie mal supportés [6, 7]. La notion d'épisodes successifs neuropsychiatrique et/ou digestive intermittente sans diagnostic retrouvé doit faire rechercher ce diagnostic [8]. Entre les accès, la présentation peut être parfaitement normale, mais certains patients présentent un retard mental ou un diagnostic de maladie psychiatrique [9-10], l'intensité des séquelles est corrélée au nombre et à la gravité des accès [11]. Les patients porteurs d'une révélation tardive d'un déficit du cycle de l'urée se présentent avec une encéphalopathie d'aggravation rapidement progressive. Le tableau clinique initial associe, invariablement, une léthargie, des vomissements, une anorexie et une irritabilité [12]. S'y associent, plutôt dans les révélations tardives, une ataxie, un comportement agressif et des troubles du comportement [13]. La présentation s'aggrave avec un niveau variable de somnolence, une ophtalmoplégie, une rigidité de décortication et de décérébration, une comitialité, une disparition du réflexe cornéen et le décès par engagement cérébral.

Certains facteurs de décompensation ont donc été clairement mis en évidence. Il s'agit d'apports protéiques inhabituels ou excessifs au cours d'un repas [14], d'un régime protidique ou hypocalorique draconien, d'une alimentation artificielle lors d'une hospitalisation [15]. Une hémorragie digestive peut constituer une charge protidique importante. Les processus protéolytiques tels que les infections bactériennes ou virales [16], les vaccinations, le postpartum [17], les traumatismes, les états de stress en général sont susceptibles de provoquer un accès hyperammoniémique. Certains médicaments comme les glucocorticoïdes ou les chimiothérapies accroissent le catabolisme protidique. Dans un nombre important de cas, l'administration de valproate de sodium a été impliquée dans la survenue ou l'aggravation d'une décompensation [18]. Ces médicaments favorisent l'aggravation du déficit du cycle de l'urée par un effet d'inhibition sur les enzymes du cycle de l'urée. Sur le plan paraclinique, l'analyse standard du liquide cérébro-spinal est normale. L'électro-encéphalogramme peut montrer des signes peu spécifiques « d'encéphalopathie métabolique ». L'imagerie cérébrale par tomodensitométrie ou IRM peut être normale ou montrer des signes de «pseudo-stroke» ou d'œdème cérébral. Pour évoquer un UCD, l'étape-clé est la mise en évidence d'une hyperammoniémie, sur sang veineux. Les conditions de prélèvements et d'acheminement doivent être rigoureusement respectées pour obtenir un dosage fiable. Au cours des décompensations aiguës des UCD, l'ammoniémie peut dépasser 1000 mol/L. La chromatographie des acides aminés plasmatiques (CAA) permet de mesurer les concentrations en acides aminés transporteurs d'ammoniac (glutamine principalement, mais aussi glycine, et alanine) et des 4 acides aminés spécifiques du cycle de l'urée que sont la citrulline, l'argininosuccinate, l'arginine et l'ornithine. Le CAA met en évidence une augmentation des acides aminés en amont et un déficit de ceux d'aval [1].

La prise en charge des UCD comprend le traitement d'urgence des décompensations aiguës hyperammoniémiques et le traitement d'entretien. En phase aiguë, le traitement d'urgence consiste à stopper l'apport naturel ou artificiel des protéines qui alimentent le pool d'acides aminés pourvoyeurs d'une hyperammoniémie. En parallèle, il est indispensable d'assurer un apport énergétique glucidolipidique suffisant pour freiner le catabolisme des protéines endogènes qui aggravent la situation en alimentant le pool d'acides aminés. Il convient également d'avoir recours à un traitement dit « épurateur » permettant ainsi de court-circuiter le cycle de l'urée, les acides aminés transporteurs d'ammoniac seront ainsi dérivés en amont du cycle de l'urée vers une voie alternative, parmi les épurateurs utilisés, le benzoate de sodium qui se conjugue à la glycine pour former l'acide hippurique non toxique qui sera excrété dans les urines, le phénylbutyrate qui se conjugue à la glutamine pour former la phénylacétylglutamine également excrétée dans les urines. Dans les cas graves avec troubles de conscience, coma, et hyperammoniémie massive, le traitement repose sur une épuration exogène par hémodialyse. Le traitement d'entretien repose principalement sur des mesures nutritionnelles. L'apport protidique naturel est limité et la tolérance protidique est évaluée pour chaque individu. Le traitement épurateur oral par phénylbutyrate de sodium, 9 à 13 g/m2 sans dépasser 20 g/j et le plus souvent en dessous de 12 g/j, permet le plus souvent d'améliorer la tolérance protidique et pourra être proposé chez certains patients adultes [19].

## Conclusion

Les UCD peuvent se révéler à tout âge, notamment chez l'adulte par des décompensations métaboliques aiguës potentiellement mortelles. L'association de signes neuropsychiatriques et digestifs doit faire évoquer le diagnostic et conduire à mesurer l'ammoniémie en urgence. En cas d'hyperammoniémie, une démarche diagnostique spécifique doit être engagée mais ne doit pas retarder la mise en route du traitement. Les UCD sont en effet des maladies traitables, dont le pronostic d'une décompensation aiguë dépend du délai de mise en route du traitement.

## Conflits d’intérêts

Les auteurs ne déclarent aucun conflit d'intérêts.
